# Methyl 4-(4-methyl­benzamido)-2-sulfamoylbenzoate

**DOI:** 10.1107/S160053680803599X

**Published:** 2008-11-08

**Authors:** Mei-Yi Wang

**Affiliations:** a College of Chemistry and Chemical Engineering, The North University for Ethnics, Yinchuan, 750021, People’s Republic of China

## Abstract

The title compound, C_16_H_16_N_2_O_5_S, is a potent new fungicide. There are two mol­ecules in the asymmetric unit which are linked by C—H⋯π inter­actions, forming a dimer. The two phenyl rings in each mol­ecules are almost coplanar, with C—N—C—C torsion angles of 177.6 (2) and −172.5 (2)°. There are inter­molecular and intra­molecular N—H⋯O hydrogen bonds in the crystal structure.

## Related literature

For the preparation and properties of substituted amides, see: Gong *et al.* (2008 ▶); Liu *et al.* (2007*a* ▶,*b* ▶); Wang *et al.* (2008 ▶).
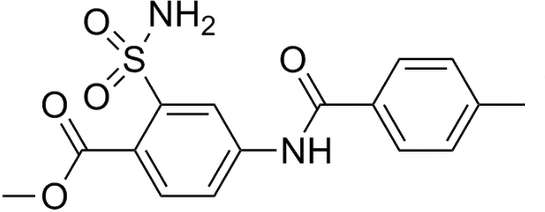


## Experimental

### 

#### Crystal data

C_16_H_16_N_2_O_5_S
*M* *_r_* = 348.37
Triclinic, 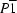

*a* = 9.1968 (16) Å
*b* = 11.078 (2) Å
*c* = 15.914 (3) Å
α = 75.894 (3)°
β = 87.124 (3)°
γ = 84.123 (3)°
*V* = 1563.7 (5) Å^3^
*Z* = 4
Mo *K*α radiation
μ = 0.24 mm^−1^
*T* = 294 (2) K
0.24 × 0.20 × 0.14 mm

#### Data collection

Bruker SMART CCD area-detector diffractometer
Absorption correction: multi-scan (*SADABS*; Sheldrick, 1996 ▶) *T*_min_ = 0.945, *T*_max_ = 0.968
8144 measured reflections
5479 independent reflections
3677 reflections with *I* > 2σ(*I*)
*R*_int_ = 0.023

#### Refinement

*R*[*F* ^2^ > 2σ(*F* ^2^)] = 0.042
*wR*(*F* ^2^) = 0.120
*S* = 1.01
5479 reflections
461 parameters
8 restraints
H atoms treated by a mixture of independent and constrained refinement
Δρ_max_ = 0.21 e Å^−3^
Δρ_min_ = −0.32 e Å^−3^

### 

Data collection: *SMART* (Bruker, 2004 ▶); cell refinement: *SAINT* (Bruker, 2004 ▶); data reduction: *SAINT*; program(s) used to solve structure: *SHELXS97* (Sheldrick, 2008 ▶); program(s) used to refine structure: *SHELXL97* (Sheldrick, 2008 ▶); molecular graphics: *SHELXTL* (Sheldrick, 2008 ▶); software used to prepare material for publication: *SHELXTL*.

## Supplementary Material

Crystal structure: contains datablocks 070704Bc, I. DOI: 10.1107/S160053680803599X/bq2101sup1.cif

Structure factors: contains datablocks I. DOI: 10.1107/S160053680803599X/bq2101Isup2.hkl

Additional supplementary materials:  crystallographic information; 3D view; checkCIF report

## Figures and Tables

**Table 1:** Hydrogen-bond geometry (Å, °)

*D*—H⋯*A*	*D*—H	H⋯*A*	*D*⋯*A*	*D*—H⋯*A*
N1—H1*A*⋯O7^i^	0.890 (10)	2.414 (15)	3.256 (3)	158 (2)
N2—H2*A*⋯O8^ii^	0.888 (10)	2.166 (12)	2.993 (3)	155 (2)
N3—H3*A*⋯O8^iii^	0.889 (10)	2.59 (2)	3.254 (3)	132 (2)
N4—H4*A*⋯O1^iii^	0.886 (10)	2.088 (11)	2.958 (3)	168 (3)
N2—H2*B*⋯O4	0.892 (10)	2.149 (18)	2.905 (3)	142 (2)
N4—H4*B*⋯O9	0.888 (10)	2.10 (2)	2.789 (3)	134 (2)
N4—H4*B*⋯O6^iv^	0.888 (10)	2.59 (2)	3.298 (3)	137 (2)
C7—H7*A*⋯*Cg*1^ii^	0.96	2.87	3.7491	148 (2)

Symmetry codes: (i) 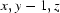; (ii) 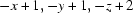; (iii) 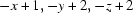; (iv) 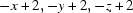. *Cg*1 is the centroid of the C17--C22 ring.

**Fig. 1.**
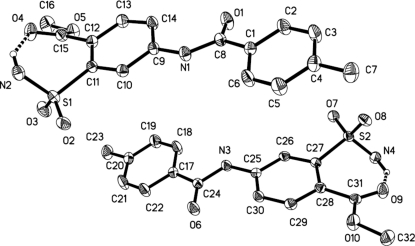
The structure of (I) with displacement ellipsoids drawn at the 30% probability level.

**Fig. 2.**
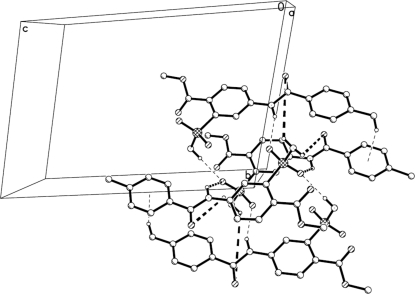
Partial packing diagram for (I). The dotted lines show the C-H···π interactions and H-bond. H atoms have been omitted for clarity.

**Fig. 3.**
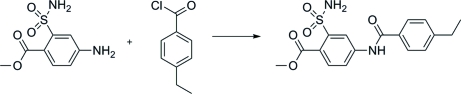
Reaction scheme.

## supplementary crystallographic information

### Comment

Amide derivative as a kind of highly bioactive compound has been studied broadly for many years. Whereas numerous references to the preparations and properties of a large variety of substituted amides exist in the literature (Liu *et al.*, 2007a). These compounds had long been used in agriculture (Liu *et al.*, 2007b) and medicine (Gong *et al.*,2008). In view of these facts and in continuation of our interest in the agriculture, we attempted to synthesize a series of amide derivatives, some of which have comparatively high fungicidal activity.

The molecular structure of title compound is showing in Fig.1. The X-ray analysis reveals that the benzene ring is planar. The carboxamide moiety is coplanar with the benzene ring [dihedral angle -1.5 (4)°]. The crystal structure is stabilized by the formation of inversion related dimers linked by C-H···π interactions (Table 1, Fig. 2).

### Experimental

The title compound was prepared according to the similar reported procedure (Wang, *et al.*, 2008). Dropwised 4-methylbenzoyl chloride (7.5 mmol) to methyl 4-amino-2-sulfamoylbenzoate (7.50 mmol) in THF (20 ml) solution, then refluxed for 4 h. Colorless single crystals suitable for X-ray diffraction were obtained by recrystallization from a mixture of ethyl acetate and petroleum ether.

### Refinement

The structure was solved by direct methods and successive Fourier difference synthesis. The H atoms bonded to C and N atoms were positioned geometrically and refined using a riding model [aromatic C—H=0.93Å, aliphatic C—H = 0.97 (2)Å, N—H=0.86Å, *U*_iso_(H) = 1.2*U*_eq_(C)].

### Crystal data

**Table:**

C_16_H_16_N_2_O_5_S	*Z* = 4
*M**_r_* = 348.37	*F*_000_ = 728
Triclinic, *P*1	*D*_x_ = 1.480 Mg m^−^^3^
Hall symbol: -P 1	Mo *K*α radiation λ = 0.71073 Å
*a* = 9.1968 (16) Å	Cell parameters from 2452 reflections
*b* = 11.078 (2) Å	θ = 2.6–25.9º
*c* = 15.914 (3) Å	µ = 0.24 mm^−^^1^
α = 75.894 (3)º	*T* = 294 (2) K
β = 87.124 (3)º	Clubbed, colorless
γ = 84.123 (3)º	0.24 × 0.20 × 0.14 mm
*V* = 1563.7 (5) Å^3^	

### Data collection

**Table:**

Bruker SMART CCD area-detector diffractometer	5479 independent reflections
Radiation source: fine-focus sealed tube	3677 reflections with *I* > 2σ(*I*)
Monochromator: graphite	*R*_int_ = 0.023
*T* = 294(2) K	θ_max_ = 25.0º
phi and ω scans	θ_min_ = 1.9º
Absorption correction: multi-scan(SADABS; Sheldrick, 1996)	*h* = −10→8
*T*_min_ = 0.945, *T*_max_ = 0.968	*k* = −13→10
8144 measured reflections	*l* = −18→18

### Refinement

**Table:**

Refinement on *F*^2^	Secondary atom site location: difference Fourier map
Least-squares matrix: full	Hydrogen site location: inferred from neighbouring sites
*R*[*F*^2^ > 2σ(*F*^2^)] = 0.042	H atoms treated by a mixture of independent and constrained refinement
*wR*(*F*^2^) = 0.120	*w* = 1/[σ^2^(*F*_o_^2^) + (0.0551*P*)^2^ + 0.5359*P*] where *P* = (*F*_o_^2^ + 2*F*_c_^2^)/3
*S* = 1.02	(Δ/σ)_max_ = 0.003
5479 reflections	Δρ_max_ = 0.22 e Å^−^^3^
461 parameters	Δρ_min_ = −0.32 e Å^−^^3^
8 restraints	Extinction correction: none
Primary atom site location: structure-invariant direct methods	

### Special details

**Table:**

Geometry. All e.s.d.'s (except the e.s.d. in the dihedral angle between two l.s. planes) are estimated using the full covariance matrix. The cell e.s.d.'s are taken into account individually in the estimation of e.s.d.'s in distances, angles and torsion angles; correlations between e.s.d.'s in cell parameters are only used when they are defined by crystal symmetry. An approximate (isotropic) treatment of cell e.s.d.'s is used for estimating e.s.d.'s involving l.s. planes.
Refinement. Refinement of *F*^2^ against ALL reflections. The weighted *R*-factor *wR* and goodness of fit *S* are based on *F*^2^, conventional *R*-factors *R* are based on *F*, with *F* set to zero for negative *F*^2^. The threshold expression of *F*^2^ > σ(*F*^2^) is used only for calculating *R*-factors(gt) *etc*. and is not relevant to the choice of reflections for refinement. *R*-factors based on *F*^2^ are statistically about twice as large as those based on *F*, and *R*- factors based on ALL data will be even larger.

### Fractional atomic coordinates and isotropic or equivalent isotropic displacement parameters (Å^2^)

**Table:**

	*x*	*y*	*z*	*U*_iso_*/*U*_eq_	
S1	0.48593 (7)	0.28763 (6)	0.70812 (4)	0.0363 (2)	
S2	0.64701 (7)	1.09651 (6)	1.08377 (4)	0.03159 (18)	
O1	0.1820 (2)	0.58510 (18)	1.01610 (12)	0.0516 (6)	
O2	0.59114 (19)	0.21940 (18)	0.76916 (12)	0.0465 (5)	
O3	0.5328 (2)	0.36685 (19)	0.62914 (12)	0.0530 (6)	
O4	0.2127 (2)	0.40235 (19)	0.58979 (12)	0.0556 (6)	
O5	0.1961 (2)	0.60599 (18)	0.58636 (12)	0.0495 (5)	
O6	0.9616 (2)	0.79515 (19)	0.78099 (12)	0.0543 (6)	
O7	0.55730 (19)	1.17378 (17)	1.01674 (11)	0.0404 (5)	
O8	0.57554 (19)	1.02353 (17)	1.15791 (11)	0.0431 (5)	
O9	0.8735 (2)	0.96320 (19)	1.21207 (12)	0.0518 (6)	
O10	0.9933 (2)	0.78427 (19)	1.20227 (12)	0.0571 (6)	
N1	0.3212 (3)	0.4194 (2)	0.98482 (14)	0.0377 (6)	
N2	0.3947 (3)	0.1860 (2)	0.68291 (16)	0.0423 (6)	
N3	0.7773 (2)	0.9255 (2)	0.81909 (14)	0.0393 (6)	
N4	0.7427 (2)	1.1888 (2)	1.11465 (16)	0.0376 (6)	
C1	0.3070 (3)	0.4445 (2)	1.13223 (16)	0.0325 (6)	
C2	0.2253 (3)	0.4934 (3)	1.19400 (18)	0.0480 (8)	
H2	0.1469	0.5530	1.1770	0.058*	
C3	0.2596 (3)	0.4544 (3)	1.28001 (18)	0.0522 (8)	
H3	0.2023	0.4876	1.3203	0.063*	
C4	0.3756 (3)	0.3677 (3)	1.30870 (17)	0.0410 (7)	
C5	0.4579 (3)	0.3219 (3)	1.24656 (19)	0.0533 (8)	
H5	0.5386	0.2646	1.2633	0.064*	
C6	0.4239 (3)	0.3587 (3)	1.16056 (18)	0.0485 (8)	
H6	0.4811	0.3249	1.1205	0.058*	
C7	0.4133 (4)	0.3275 (3)	1.40279 (18)	0.0558 (9)	
H7A	0.3940	0.2419	1.4253	0.084*	
H7B	0.3551	0.3793	1.4345	0.084*	
H7C	0.5150	0.3354	1.4087	0.084*	
C8	0.2647 (3)	0.4900 (2)	1.04024 (16)	0.0340 (6)	
C9	0.2944 (3)	0.4394 (2)	0.89600 (16)	0.0324 (6)	
C10	0.3841 (3)	0.3679 (2)	0.84935 (16)	0.0334 (6)	
H10	0.4578	0.3106	0.8773	0.040*	
C11	0.3642 (3)	0.3814 (2)	0.76245 (16)	0.0312 (6)	
C12	0.2557 (3)	0.4688 (2)	0.71804 (16)	0.0345 (6)	
C13	0.1686 (3)	0.5392 (3)	0.76606 (17)	0.0407 (7)	
H13	0.0970	0.5987	0.7379	0.049*	
C14	0.1844 (3)	0.5242 (3)	0.85342 (17)	0.0400 (7)	
H14	0.1218	0.5706	0.8839	0.048*	
C15	0.2222 (3)	0.4858 (3)	0.62516 (17)	0.0380 (7)	
C16	0.1575 (4)	0.6338 (3)	0.49659 (19)	0.0649 (10)	
H16A	0.2336	0.5978	0.4642	0.097*	
H16B	0.1455	0.7227	0.4740	0.097*	
H16C	0.0675	0.5993	0.4918	0.097*	
C17	0.8123 (3)	0.9163 (3)	0.66853 (17)	0.0359 (6)	
C18	0.7012 (3)	1.0074 (3)	0.63916 (19)	0.0544 (9)	
H18	0.6432	1.0407	0.6791	0.065*	
C19	0.6733 (3)	1.0506 (3)	0.55252 (19)	0.0556 (9)	
H19	0.5980	1.1132	0.5353	0.067*	
C20	0.7543 (3)	1.0035 (3)	0.49037 (18)	0.0427 (7)	
C21	0.8621 (4)	0.9087 (3)	0.51993 (19)	0.0571 (9)	
H21	0.9166	0.8727	0.4800	0.069*	
C22	0.8920 (3)	0.8655 (3)	0.60655 (18)	0.0509 (8)	
H22	0.9661	0.8017	0.6239	0.061*	
C23	0.7282 (4)	1.0553 (3)	0.39532 (18)	0.0587 (9)	
H23A	0.6304	1.0431	0.3829	0.088*	
H23B	0.7967	1.0128	0.3623	0.088*	
H23C	0.7408	1.1430	0.3801	0.088*	
C24	0.8570 (3)	0.8714 (3)	0.76026 (17)	0.0356 (6)	
C25	0.8069 (3)	0.9098 (2)	0.90663 (16)	0.0318 (6)	
C26	0.7350 (3)	0.9965 (2)	0.94941 (16)	0.0318 (6)	
H26	0.6692	1.0599	0.9195	0.038*	
C27	0.7607 (3)	0.9889 (2)	1.03508 (15)	0.0283 (6)	
C28	0.8636 (3)	0.8951 (2)	1.08109 (16)	0.0309 (6)	
C29	0.9298 (3)	0.8095 (2)	1.03729 (17)	0.0360 (6)	
H29	0.9963	0.7461	1.0665	0.043*	
C30	0.9014 (3)	0.8142 (2)	0.95255 (16)	0.0355 (6)	
H30	0.9457	0.7531	0.9262	0.043*	
C31	0.9073 (3)	0.8870 (2)	1.17093 (16)	0.0329 (6)	
C32	1.0496 (3)	0.7699 (3)	1.28726 (18)	0.0553 (9)	
H32A	1.0717	0.8498	1.2942	0.083*	
H32B	1.1369	0.7134	1.2940	0.083*	
H32C	0.9777	0.7371	1.3303	0.083*	
H1A	0.382 (3)	0.3537 (18)	1.0090 (17)	0.056 (9)*	
H2A	0.374 (3)	0.1235 (19)	0.7272 (12)	0.051 (9)*	
H2B	0.319 (2)	0.222 (2)	0.6504 (14)	0.059 (10)*	
H3A	0.700 (2)	0.979 (2)	0.8004 (17)	0.055 (9)*	
H4A	0.775 (3)	1.2487 (19)	1.0724 (12)	0.051 (9)*	
H4B	0.806 (2)	1.151 (2)	1.1556 (13)	0.062 (10)*	

### Atomic displacement parameters (Å^2^)

**Table:**

	*U*^11^	*U*^22^	*U*^33^	*U*^12^	*U*^13^	*U*^23^
S1	0.0364 (4)	0.0364 (4)	0.0364 (4)	0.0022 (3)	0.0045 (3)	−0.0123 (3)
S2	0.0301 (4)	0.0323 (4)	0.0305 (4)	0.0073 (3)	−0.0017 (3)	−0.0079 (3)
O1	0.0721 (14)	0.0415 (12)	0.0356 (11)	0.0238 (11)	−0.0059 (10)	−0.0095 (9)
O2	0.0377 (11)	0.0493 (12)	0.0521 (12)	0.0131 (9)	−0.0062 (9)	−0.0173 (10)
O3	0.0604 (13)	0.0513 (13)	0.0464 (12)	−0.0103 (11)	0.0193 (10)	−0.0120 (10)
O4	0.0787 (16)	0.0478 (13)	0.0415 (12)	0.0046 (12)	−0.0141 (11)	−0.0149 (10)
O5	0.0683 (14)	0.0400 (12)	0.0349 (11)	0.0076 (10)	−0.0095 (10)	−0.0024 (9)
O6	0.0574 (13)	0.0596 (14)	0.0436 (12)	0.0261 (11)	−0.0084 (10)	−0.0197 (11)
O7	0.0395 (11)	0.0411 (11)	0.0379 (11)	0.0156 (9)	−0.0102 (8)	−0.0101 (9)
O8	0.0397 (11)	0.0453 (12)	0.0382 (11)	0.0025 (9)	0.0088 (9)	−0.0030 (9)
O9	0.0670 (14)	0.0497 (13)	0.0380 (11)	0.0193 (11)	−0.0147 (10)	−0.0166 (10)
O10	0.0750 (15)	0.0503 (13)	0.0406 (12)	0.0300 (12)	−0.0232 (11)	−0.0106 (10)
N1	0.0463 (14)	0.0334 (13)	0.0311 (13)	0.0108 (11)	−0.0048 (10)	−0.0087 (10)
N2	0.0500 (16)	0.0340 (14)	0.0429 (15)	0.0016 (12)	−0.0002 (13)	−0.0115 (12)
N3	0.0349 (13)	0.0495 (15)	0.0336 (13)	0.0132 (12)	−0.0062 (10)	−0.0162 (11)
N4	0.0408 (14)	0.0331 (14)	0.0387 (14)	0.0032 (11)	−0.0059 (12)	−0.0099 (11)
C1	0.0370 (15)	0.0282 (14)	0.0319 (14)	−0.0013 (12)	−0.0018 (12)	−0.0070 (11)
C2	0.0487 (18)	0.0529 (19)	0.0390 (17)	0.0212 (15)	−0.0050 (14)	−0.0144 (14)
C3	0.064 (2)	0.057 (2)	0.0338 (16)	0.0158 (17)	0.0003 (14)	−0.0156 (14)
C4	0.0524 (18)	0.0353 (16)	0.0341 (15)	−0.0038 (14)	−0.0037 (13)	−0.0057 (13)
C5	0.059 (2)	0.0521 (19)	0.0432 (18)	0.0233 (16)	−0.0097 (15)	−0.0095 (15)
C6	0.0571 (19)	0.0495 (18)	0.0363 (16)	0.0185 (15)	−0.0016 (14)	−0.0150 (14)
C7	0.074 (2)	0.052 (2)	0.0383 (17)	0.0018 (17)	−0.0105 (16)	−0.0059 (15)
C8	0.0386 (15)	0.0307 (15)	0.0325 (14)	−0.0015 (13)	0.0013 (12)	−0.0084 (12)
C9	0.0374 (15)	0.0290 (14)	0.0307 (14)	−0.0010 (12)	−0.0008 (12)	−0.0081 (11)
C10	0.0351 (15)	0.0279 (14)	0.0361 (15)	0.0014 (12)	−0.0022 (12)	−0.0073 (12)
C11	0.0325 (14)	0.0285 (14)	0.0325 (14)	0.0000 (12)	−0.0002 (11)	−0.0086 (11)
C12	0.0388 (15)	0.0324 (15)	0.0316 (14)	0.0004 (12)	−0.0036 (12)	−0.0071 (12)
C13	0.0416 (16)	0.0408 (17)	0.0379 (16)	0.0123 (13)	−0.0122 (13)	−0.0106 (13)
C14	0.0426 (17)	0.0390 (16)	0.0394 (16)	0.0094 (13)	−0.0034 (13)	−0.0161 (13)
C15	0.0367 (16)	0.0418 (17)	0.0330 (15)	0.0038 (13)	−0.0025 (12)	−0.0070 (13)
C16	0.086 (3)	0.065 (2)	0.0357 (18)	0.008 (2)	−0.0110 (17)	−0.0007 (16)
C17	0.0353 (15)	0.0380 (16)	0.0372 (15)	−0.0008 (13)	−0.0023 (12)	−0.0156 (12)
C18	0.059 (2)	0.066 (2)	0.0393 (17)	0.0270 (17)	−0.0078 (15)	−0.0258 (16)
C19	0.058 (2)	0.061 (2)	0.0457 (19)	0.0245 (17)	−0.0130 (15)	−0.0193 (16)
C20	0.0486 (18)	0.0449 (17)	0.0388 (16)	−0.0052 (15)	−0.0019 (13)	−0.0175 (14)
C21	0.069 (2)	0.063 (2)	0.0392 (18)	0.0207 (18)	0.0022 (15)	−0.0224 (16)
C22	0.0561 (19)	0.0534 (19)	0.0408 (17)	0.0197 (16)	−0.0012 (14)	−0.0171 (15)
C23	0.074 (2)	0.064 (2)	0.0374 (17)	0.0037 (19)	−0.0082 (16)	−0.0148 (16)
C24	0.0330 (15)	0.0386 (16)	0.0371 (15)	0.0042 (13)	−0.0043 (12)	−0.0154 (13)
C25	0.0278 (14)	0.0353 (15)	0.0339 (14)	−0.0016 (12)	−0.0014 (11)	−0.0121 (12)
C26	0.0270 (14)	0.0320 (15)	0.0344 (15)	0.0048 (11)	−0.0037 (11)	−0.0066 (12)
C27	0.0279 (13)	0.0247 (13)	0.0309 (14)	−0.0005 (11)	0.0008 (11)	−0.0049 (11)
C28	0.0295 (14)	0.0308 (14)	0.0308 (14)	−0.0002 (12)	−0.0002 (11)	−0.0050 (11)
C29	0.0356 (15)	0.0324 (15)	0.0365 (15)	0.0080 (13)	−0.0046 (12)	−0.0051 (12)
C30	0.0361 (15)	0.0345 (15)	0.0353 (15)	0.0058 (12)	0.0001 (12)	−0.0110 (12)
C31	0.0287 (14)	0.0359 (16)	0.0310 (14)	−0.0011 (12)	0.0008 (11)	−0.0030 (12)
C32	0.062 (2)	0.060 (2)	0.0378 (17)	0.0126 (17)	−0.0176 (15)	−0.0043 (15)

### Geometric parameters (Å, °)

**Table:**

S1—O3	1.422 (2)	C9—C14	1.388 (3)
S1—O2	1.4275 (19)	C9—C10	1.397 (3)
S1—N2	1.606 (2)	C10—C11	1.374 (3)
S1—C11	1.784 (2)	C10—H10	0.9300
S2—O8	1.4296 (19)	C11—C12	1.405 (3)
S2—O7	1.4318 (17)	C12—C13	1.391 (4)
S2—N4	1.592 (2)	C12—C15	1.488 (4)
S2—C27	1.797 (2)	C13—C14	1.373 (3)
O1—C8	1.226 (3)	C13—H13	0.9300
O4—C15	1.206 (3)	C14—H14	0.9300
O5—C15	1.327 (3)	C16—H16A	0.9600
O5—C16	1.441 (3)	C16—H16B	0.9600
O6—C24	1.216 (3)	C16—H16C	0.9600
O9—C31	1.197 (3)	C17—C18	1.374 (4)
O10—C31	1.322 (3)	C17—C22	1.393 (4)
O10—C32	1.439 (3)	C17—C24	1.487 (4)
N1—C8	1.366 (3)	C18—C19	1.373 (4)
N1—C9	1.407 (3)	C18—H18	0.9300
N1—H1A	0.890 (10)	C19—C20	1.379 (4)
N2—H2A	0.888 (10)	C19—H19	0.9300
N2—H2B	0.892 (10)	C20—C21	1.379 (4)
N3—C24	1.374 (3)	C20—C23	1.503 (4)
N3—C25	1.398 (3)	C21—C22	1.376 (4)
N3—H3A	0.889 (10)	C21—H21	0.9300
N4—H4A	0.886 (10)	C22—H22	0.9300
N4—H4B	0.888 (10)	C23—H23A	0.9600
C1—C6	1.376 (4)	C23—H23B	0.9600
C1—C2	1.387 (3)	C23—H23C	0.9600
C1—C8	1.485 (3)	C25—C30	1.382 (3)
C2—C3	1.373 (4)	C25—C26	1.402 (3)
C2—H2	0.9300	C26—C27	1.376 (3)
C3—C4	1.376 (4)	C26—H26	0.9300
C3—H3	0.9300	C27—C28	1.417 (3)
C4—C5	1.379 (4)	C28—C29	1.384 (3)
C4—C7	1.502 (4)	C28—C31	1.484 (3)
C5—C6	1.372 (4)	C29—C30	1.374 (3)
C5—H5	0.9300	C29—H29	0.9300
C6—H6	0.9300	C30—H30	0.9300
C7—H7A	0.9600	C32—H32A	0.9600
C7—H7B	0.9600	C32—H32B	0.9600
C7—H7C	0.9600	C32—H32C	0.9600
			
O3—S1—O2	119.98 (13)	C12—C13—H13	118.8
O3—S1—N2	106.99 (13)	C13—C14—C9	119.7 (2)
O2—S1—N2	106.49 (13)	C13—C14—H14	120.2
O3—S1—C11	107.35 (12)	C9—C14—H14	120.2
O2—S1—C11	107.47 (11)	O4—C15—O5	123.6 (2)
N2—S1—C11	108.08 (12)	O4—C15—C12	125.2 (3)
O8—S2—O7	117.81 (11)	O5—C15—C12	111.1 (2)
O8—S2—N4	108.82 (12)	O5—C16—H16A	109.5
O7—S2—N4	105.66 (12)	O5—C16—H16B	109.5
O8—S2—C27	106.89 (11)	H16A—C16—H16B	109.5
O7—S2—C27	106.71 (11)	O5—C16—H16C	109.5
N4—S2—C27	110.93 (12)	H16A—C16—H16C	109.5
C15—O5—C16	116.1 (2)	H16B—C16—H16C	109.5
C31—O10—C32	117.0 (2)	C18—C17—C22	116.9 (3)
C8—N1—C9	127.6 (2)	C18—C17—C24	125.2 (2)
C8—N1—H1A	114.6 (19)	C22—C17—C24	117.8 (2)
C9—N1—H1A	117.9 (19)	C19—C18—C17	121.9 (3)
S1—N2—H2A	114.3 (18)	C19—C18—H18	119.1
S1—N2—H2B	111.7 (18)	C17—C18—H18	119.1
H2A—N2—H2B	114.7 (16)	C18—C19—C20	121.7 (3)
C24—N3—C25	127.0 (2)	C18—C19—H19	119.2
C24—N3—H3A	118.2 (19)	C20—C19—H19	119.2
C25—N3—H3A	114.8 (19)	C21—C20—C19	116.5 (3)
S2—N4—H4A	114.9 (17)	C21—C20—C23	121.9 (3)
S2—N4—H4B	114.4 (18)	C19—C20—C23	121.5 (3)
H4A—N4—H4B	116.4 (16)	C22—C21—C20	122.3 (3)
C6—C1—C2	117.4 (2)	C22—C21—H21	118.9
C6—C1—C8	124.5 (2)	C20—C21—H21	118.9
C2—C1—C8	118.1 (2)	C21—C22—C17	120.6 (3)
C3—C2—C1	120.5 (3)	C21—C22—H22	119.7
C3—C2—H2	119.7	C17—C22—H22	119.7
C1—C2—H2	119.7	C20—C23—H23A	109.5
C2—C3—C4	122.3 (3)	C20—C23—H23B	109.5
C2—C3—H3	118.9	H23A—C23—H23B	109.5
C4—C3—H3	118.9	C20—C23—H23C	109.5
C3—C4—C5	116.7 (3)	H23A—C23—H23C	109.5
C3—C4—C7	121.8 (3)	H23B—C23—H23C	109.5
C5—C4—C7	121.5 (3)	O6—C24—N3	122.3 (2)
C6—C5—C4	121.6 (3)	O6—C24—C17	121.7 (2)
C6—C5—H5	119.2	N3—C24—C17	116.0 (2)
C4—C5—H5	119.2	C30—C25—N3	123.7 (2)
C5—C6—C1	121.5 (3)	C30—C25—C26	118.9 (2)
C5—C6—H6	119.3	N3—C25—C26	117.3 (2)
C1—C6—H6	119.3	C27—C26—C25	120.8 (2)
C4—C7—H7A	109.5	C27—C26—H26	119.6
C4—C7—H7B	109.5	C25—C26—H26	119.6
H7A—C7—H7B	109.5	C26—C27—C28	120.5 (2)
C4—C7—H7C	109.5	C26—C27—S2	115.51 (18)
H7A—C7—H7C	109.5	C28—C27—S2	123.87 (18)
H7B—C7—H7C	109.5	C29—C28—C27	117.0 (2)
O1—C8—N1	122.2 (2)	C29—C28—C31	119.0 (2)
O1—C8—C1	121.4 (2)	C27—C28—C31	123.9 (2)
N1—C8—C1	116.4 (2)	C30—C29—C28	122.8 (2)
C14—C9—C10	119.1 (2)	C30—C29—H29	118.6
C14—C9—N1	123.9 (2)	C28—C29—H29	118.6
C10—C9—N1	117.0 (2)	C29—C30—C25	119.8 (2)
C11—C10—C9	120.5 (2)	C29—C30—H30	120.1
C11—C10—H10	119.7	C25—C30—H30	120.1
C9—C10—H10	119.7	O9—C31—O10	121.9 (2)
C10—C11—C12	121.0 (2)	O9—C31—C28	126.0 (2)
C10—C11—S1	117.22 (19)	O10—C31—C28	112.1 (2)
C12—C11—S1	121.70 (19)	O10—C32—H32A	109.5
C13—C12—C11	117.1 (2)	O10—C32—H32B	109.5
C13—C12—C15	118.1 (2)	H32A—C32—H32B	109.5
C11—C12—C15	124.7 (2)	O10—C32—H32C	109.5
C14—C13—C12	122.5 (2)	H32A—C32—H32C	109.5
C14—C13—H13	118.8	H32B—C32—H32C	109.5
			
C6—C1—C2—C3	−1.4 (5)	C22—C17—C18—C19	−2.7 (5)
C8—C1—C2—C3	179.8 (3)	C24—C17—C18—C19	175.1 (3)
C1—C2—C3—C4	0.9 (5)	C17—C18—C19—C20	0.9 (5)
C2—C3—C4—C5	0.5 (5)	C18—C19—C20—C21	1.6 (5)
C2—C3—C4—C7	179.0 (3)	C18—C19—C20—C23	−177.0 (3)
C3—C4—C5—C6	−1.4 (5)	C19—C20—C21—C22	−2.2 (5)
C7—C4—C5—C6	−179.8 (3)	C23—C20—C21—C22	176.4 (3)
C4—C5—C6—C1	0.9 (5)	C20—C21—C22—C17	0.4 (5)
C2—C1—C6—C5	0.5 (5)	C18—C17—C22—C21	2.1 (5)
C8—C1—C6—C5	179.2 (3)	C24—C17—C22—C21	−175.9 (3)
C9—N1—C8—O1	−1.5 (4)	C25—N3—C24—O6	4.5 (5)
C9—N1—C8—C1	177.6 (2)	C25—N3—C24—C17	−172.5 (2)
C6—C1—C8—O1	−163.4 (3)	C18—C17—C24—O6	−175.9 (3)
C2—C1—C8—O1	15.2 (4)	C22—C17—C24—O6	1.8 (4)
C6—C1—C8—N1	17.4 (4)	C18—C17—C24—N3	1.1 (4)
C2—C1—C8—N1	−164.0 (3)	C22—C17—C24—N3	178.8 (3)
C8—N1—C9—C14	−11.7 (4)	C24—N3—C25—C30	−16.3 (4)
C8—N1—C9—C10	169.0 (3)	C24—N3—C25—C26	164.2 (3)
C14—C9—C10—C11	0.2 (4)	C30—C25—C26—C27	1.8 (4)
N1—C9—C10—C11	179.6 (2)	N3—C25—C26—C27	−178.7 (2)
C9—C10—C11—C12	1.3 (4)	C25—C26—C27—C28	1.7 (4)
C9—C10—C11—S1	179.0 (2)	C25—C26—C27—S2	−173.9 (2)
O3—S1—C11—C10	−134.1 (2)	O8—S2—C27—C26	123.5 (2)
O2—S1—C11—C10	−3.8 (3)	O7—S2—C27—C26	−3.4 (2)
N2—S1—C11—C10	110.8 (2)	N4—S2—C27—C26	−118.0 (2)
O3—S1—C11—C12	43.6 (3)	O8—S2—C27—C28	−52.0 (2)
O2—S1—C11—C12	174.0 (2)	O7—S2—C27—C28	−178.9 (2)
N2—S1—C11—C12	−71.5 (2)	N4—S2—C27—C28	66.5 (2)
C10—C11—C12—C13	−0.9 (4)	C26—C27—C28—C29	−3.2 (4)
S1—C11—C12—C13	−178.6 (2)	S2—C27—C28—C29	172.1 (2)
C10—C11—C12—C15	−177.1 (3)	C26—C27—C28—C31	174.4 (2)
S1—C11—C12—C15	5.2 (4)	S2—C27—C28—C31	−10.2 (4)
C11—C12—C13—C14	−1.0 (4)	C27—C28—C29—C30	1.2 (4)
C15—C12—C13—C14	175.5 (3)	C31—C28—C29—C30	−176.5 (2)
C12—C13—C14—C9	2.5 (4)	C28—C29—C30—C25	2.3 (4)
C10—C9—C14—C13	−2.1 (4)	N3—C25—C30—C29	176.7 (3)
N1—C9—C14—C13	178.6 (3)	C26—C25—C30—C29	−3.7 (4)
C16—O5—C15—O4	−1.3 (4)	C32—O10—C31—O9	−2.3 (4)
C16—O5—C15—C12	−178.0 (2)	C32—O10—C31—C28	176.5 (2)
C13—C12—C15—O4	−132.5 (3)	C29—C28—C31—O9	169.9 (3)
C11—C12—C15—O4	43.7 (4)	C27—C28—C31—O9	−7.7 (4)
C13—C12—C15—O5	44.2 (4)	C29—C28—C31—O10	−8.9 (4)
C11—C12—C15—O5	−139.6 (3)	C27—C28—C31—O10	173.6 (2)

### Hydrogen-bond geometry (Å, °)

**Table:**

*D*—H···*A*	*D*—H	H···*A*	*D*···*A*	*D*—H···*A*
N1—H1A···O7^i^	0.890 (10)	2.414 (15)	3.256 (3)	158 (2)
N2—H2A···O8^ii^	0.888 (10)	2.166 (12)	2.993 (3)	155 (2)
N3—H3A···O8^iii^	0.889 (10)	2.59 (2)	3.254 (3)	132 (2)
N4—H4A···O1^iii^	0.886 (10)	2.088 (11)	2.958 (3)	168 (3)
N2—H2B···O4	0.892 (10)	2.149 (18)	2.905 (3)	142 (2)
N4—H4B···O9	0.888 (10)	2.10 (2)	2.789 (3)	134 (2)
N4—H4B···O6^iv^	0.888 (10)	2.59 (2)	3.298 (3)	137 (2)
C7—H7A···Cg1^ii^	0.96	2.87	3.7491	148 (2)

Symmetry codes: (i) *x*, *y*−1, *z*; (ii) −*x*+1, −*y*+1, −*z*+2; (iii) −*x*+1, −*y*+2, −*z*+2; (iv) −*x*+2, −*y*+2, −*z*+2.
